# Electrically-Evoked Frequency-Following Response (EFFR) in the Auditory Brainstem of Guinea Pigs

**DOI:** 10.1371/journal.pone.0106719

**Published:** 2014-09-22

**Authors:** Wenxin He, Xiuyong Ding, Ruxiang Zhang, Jing Chen, Daoxing Zhang, Xihong Wu

**Affiliations:** 1 Speech and Hearing Research Center, and Key Laboratory of Machine Perception (Ministry of Education), Peking University, Beijing, People's Republic of China; 2 Department of Otorhinolaryngology Head and Neck Surgery, Beijing Friendship Hospital, Capital Medical University, Beijing, People's Republic of China; University of California, Irvine, United States of America

## Abstract

It is still a difficult clinical issue to decide whether a patient is a suitable candidate for a cochlear implant and to plan postoperative rehabilitation, especially for some special cases, such as auditory neuropathy. A partial solution to these problems is to preoperatively evaluate the functional integrity of the auditory neural pathways. For evaluating the strength of phase-locking of auditory neurons, which was not reflected in previous methods using electrically evoked auditory brainstem response (EABR), a new method for recording phase-locking related auditory responses to electrical stimulation, called the electrically evoked frequency-following response (EFFR), was developed and evaluated using guinea pigs. The main objective was to assess feasibility of the method by testing whether the recorded signals reflected auditory neural responses or artifacts. The results showed the following: 1) the recorded signals were evoked by neuron responses rather than by artifact; 2) responses evoked by periodic signals were significantly higher than those evoked by the white noise; 3) the latency of the responses fell in the expected range; 4) the responses decreased significantly after death of the guinea pigs; and 5) the responses decreased significantly when the animal was replaced by an electrical resistance. All of these results suggest the method was valid. Recording obtained using complex tones with a missing fundamental component and using pure tones with various frequencies were consistent with those obtained using acoustic stimulation in previous studies.

## Introduction

Three main types of method for evaluating auditory functions, have been widely used in research and in the clinic: audiological testing, cochlear imaging, and measurement of auditory evoked potentials (AEPs) [1]. AEPs can provide objectively information about auditory function and thus help to guide further improvements in implant technology [2]. There are a number of clearly defined clinical uses of AEP, such as electrocochleography (ECoChG) and auditory brainstem response (ABR). The ABR is currently the most popular AEP for hearing screening in clinical situations [1].

The ABR is a series of vertex-positive waves that occur within 15 ms of the onset of a click stimulus in human adults [3]. Although seven peaks are often seen, only waves I to V are evaluated in most instances. The sources of the five wave components are generally interpreted as follows: wave I comes from the cochlear action potential (CAP) and the distal portion of the eighth nerve; wave II represents the responses of proximal portion of the eighth nerve; wave III comes from the cochlear nuclei; and waves IV and V come from the superior olive, lateral lemniscus and inferior colliculus [4]. ABRs were first recorded in human subjects in 1970 by Jewett and Williston [5], and were subsequently recorded in cats [6], guinea pigs [7], and monkeys [8], among other animals. The ABR has been clinically used to diagnose retro-cochlear lesions such as acoustic neuroma [9], and it is also one of the most important methods used for universal newborn hearing screening [10].

Since the stimulation is acoustic, conventional ABRs testing is not suitable for screening the cochlear implantation. It has been reported that many hearing-impaired children with auditory neuropathy had grossly abnormal ABRs but showed excellent results with cochlear implantation [11]. In order to solve this problem, methods have been developed for measuring evoked responses to electrical stimulation. Starr and Brackmann reported the first measurements of electrically evoked auditory brainstem responses (EABRs) in human cochlear implant users in 1979 [12]. Several years later, in 1983, EABRs were introduced as a clinical tool for estimating auditory nerve survival by Simmons *et al.* [13]. Kileny and Zwolan suggested that the EABRs provided an effective preoperative assessment tool for cochlear implantation [14]. However, it was also reported that even if no EABR could be recorded, cochlear implantation sometimes provided measurable benefit for people [15], indicating that EABR does not fully reflect the residual auditory capability of the severely hearing-impaired person.

In all previous EABRs studies, the stimuli were electric clicks, which lack periodic features to elicite phase-locking responses. However, the phase-locking response is one of the most significant characteristic of the auditory system and it plays a role in many aspects of auditory perception [16]. Additionally, it was reported by Evans “At low stimulus intensities, a tone can produce significant phase-locking even though the mean firing rate is not increased. Tuning curves based on a criterion of a certain degree of phase-locking are similar to those based on an increase in firing rate, although for the above reason they may be more sensitive by 20 dB or so” [17], indicating phase-locking responses could be more sensitive than the responses recorded by the current EABRs. To test the phase-locking response of auditory system to electrical stimuli, a novel method named the electrically evoked frequency-following response (EFFR) was developed and evaluated in this study.

It is generally agreed that the human scalp-recorded frequency-following response (FFR) reflects phase-locked activity in a population of neural elements in the rostral brainstem [18], [19]. Although it is difficult to determine the exact neural generators of the FFR, several lines of evidence suggest that FFRs originate in the cochlear nucleus (CN), inferior colliculus (IC), and/or the lateral lemniscuses (LL) [20]. FFRs have been used in studies of the sound sensation in animals and speech perception in humans [21]. These studies have revealed that some critical acoustic properties of sounds are represented in subcortical auditory structures with considerably temporal and spectral precision [22]–[24].

Based on the characteristics of the FFR, we hypothesized that EFFRs could provide a method for evaluating the strength of phase-locking responses in the auditory system. Although EABRs have been widely used both in animals and humans, we are not aware of previous research on EFFRs. This article assesses whether EFFR can be recorded in the guinea pigs and whether the measured responses reflect genuine neural responses or artifacts.

Previous studies have provided several methods for assessing whether a recorded response was evoked by neural activity or by stimulus artifacts [25]–[28]. Those methods were adopted here. In Experiment I, the recorded amplitude was measured as a function of the stimulus amplitude (input-output function). It is assumed that the input-output function is linear for artifact but non-linear for neural responses [1]. In Experiment II, we examined the relative amplitude of the responses evoked by pure tones and white noise. FFRs to acoustic stimuli can only be recorded by averaging the responses to periodic stimuli. We assessed whether this was also true for the EFFR. In Experiment III, a signal whose amplitude was weighted by a Hanning window was used to estimate the latency of the response. For artifacts, this would be very short, while for neural responses a latency should exist due to the time required for the nerve conduction [29]–[31]. In the Experiment IV, we measured the relative amplitude of the EFFRs as a function of the time around the death. Artifacts would be expected to persist after death, whereas neural responses should cease rapidly after death [32]. Finally, in Experiment V we measured EFFR responses when an electrical resistance was used to replace guinea pigs, if neural responses dominate, then responses should be much lower for the resistance than for a guinea pig.

After using the experiments described above to confirm that the EFFRs reflected neural responses, we explored the properties of EFFRs in two other experiments. In Experiment VI, harmonic complex tones without a component at the fundamental frequency (F0) were used as signals to assess whether the EFFR contains a component at F0, as has been found for the acoustically-evoked FFR. In Experiment VII, the frequency of a pure tone was manipulated to examine the relative amplitude of EFFR as a function of frequency.

## Materials and Methods

This study was carried out in accordance with the Guidelines of Beijing Laboratory Animal Center, and with the Policies on the Use of Animals and Humans in Neuroscience Research approved by the Society for Neuroscience (2006). The protocol was approved by the Animal Care & Welfare Committee, Institute of Materia Medica, Chinese Academy of Medical Sciences (CAMS) and Peking Union Medical College (PUMC) (Permit Number: 002477). The whole surgery was performed under chloral hydrate anesthesia, and all efforts were made to minimize suffering.

### Animal preparation

Thirty seven young adult albino guinea pigs (twenty male and seventeen female, weight 500 g ±96 g) were provided by the laboratory animal center of the Institute of Materia Medica, CAMS & PUMC. Thirteen of them were used in a pilot experiment, and the other twenty four were divided to three groups with eight guinea pigs per group: group 1 was used for the Experiment I, group 2 for Experiments II–VII, and group 3 for the experiment described in the section “Discussion”. Please notice that we used albino guinea pigs rather than typical guinea pigs, because: 1) In the near future, we will implement the EFFR experiments not only on the normal hearing guinea pigs but also the hearing loss ones. Previous studies showed that the albino guinea pigs were easier to be deafening than the typical ones [33]; 2) Previous studies suggested that cochlear action potential thresholds [34] and ABRs [35] are similar between pigmented and albino guinea pigs.

All the guinea pigs were fed in the tray type cages with sawdust as bedding material. Every four of them were fed in one cage, where water were supplied by a lick type bottle and fodder were supplied by a square groove. Light/dark cycle was formed by turning on the sunlight lamp at 8 AM and off at 8 PM.

The guinea pigs were anesthetized with 10% chloral hydrate (400 mg/kg, ip) at the beginning of the experiment, and the state of anesthesia was monitored by paw reflex and maintained throughout the experiments by supplemental injection of the same anesthetic (200 mg/kg, ip). A heat preservation cushion and cover were used to maintain the body temperature of the guinea pigs after anesthesia. The temperature of the experimental room was kept around 23 degrees centigrade and the relative air humidity was kept at approximately 44%. The guinea pig was monitored to ensure that the breath and heartbeat were normal.

Chloral hydrate was injected intraperitoneally to ensure rapid absorption and caused physiological sleep with nearly no apparent side effects. It may cause nausea or vomiting, but these adverse effects were not seen in all the 37 guinea pigs of the experiments.

### Experimental platform

An 8-channel digital stimulator (DS8000, World Precision Instruments Ltd) was used for generating the experimental electrical stimuli signals in digital form. A biological linear stimulus isolator (DLS100, World Precision Instruments Ltd) was used to convert the digital signal to analog form. Isolation was achieved with an optical isolator and a DC-to-DC power converter. An evoked-potential instrument (Neuro-MEP-4, Neurosoft Ltd) was used to record the response potentials.

The guinea pig was placed on the dissecting table and the osseous external acoustic meatus of the stimulation ear was exposed by carefully removing the skin and subcutaneous tissue around the meatus. Then the round window niche was exposed.

**Figure 1:**
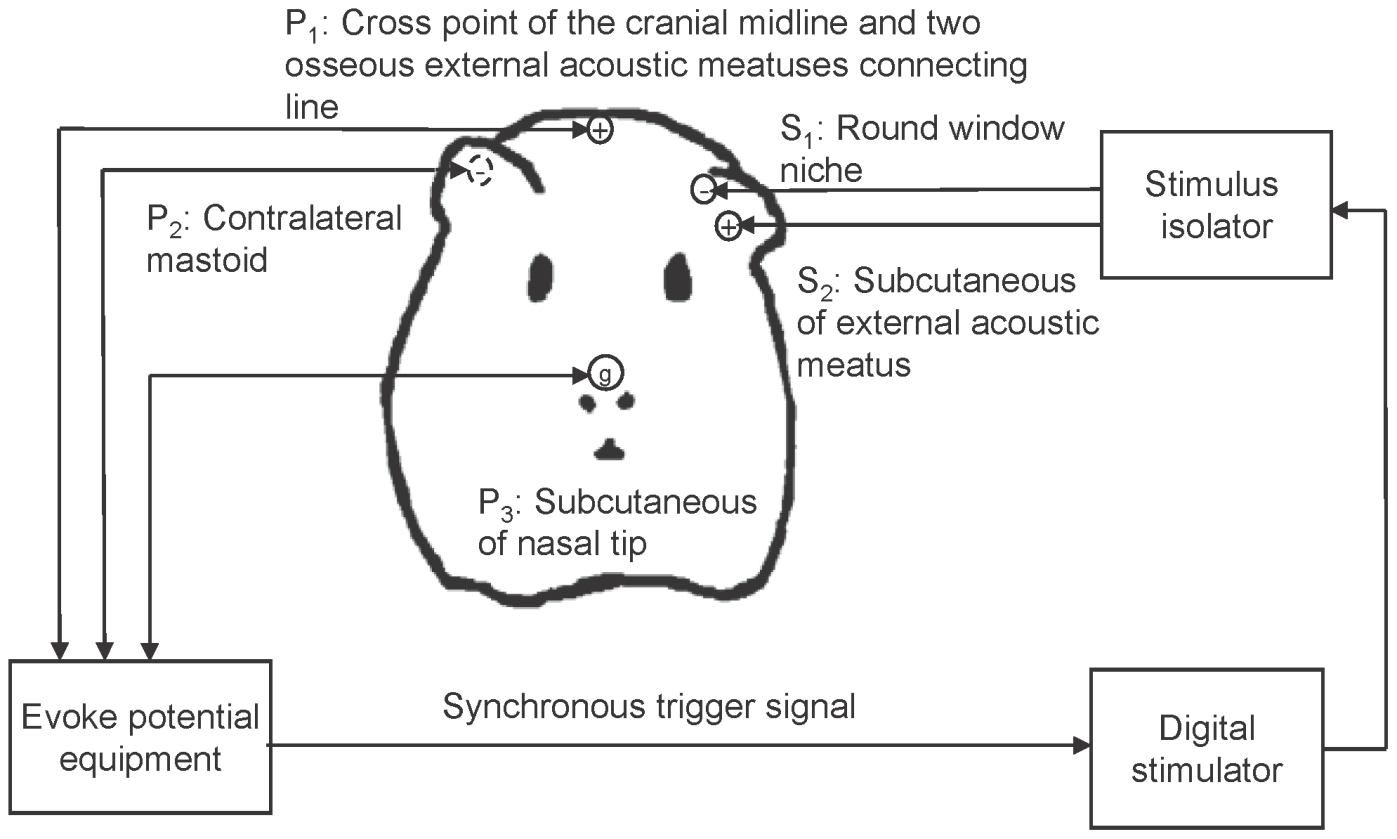
Diagram of the experimental set up.

A stimulating ball electrode made from Platinum was placed on the round window niche and the ground electrode was placed in the subcutaneous tissue of the external auditory meatus. Three recording electrodes were positioned as follows: the active electrode was placed at the cross point of the cranial midline and the line connecting the two osseous external auditory meatuses, the reference electrode was placed at the contralateral mastoid, and the ground electrode was placed at the nasal tip. A diagram of the experimental platform set up is shown in Fig. 1.

### Experimental procedure

#### 1) Pilot experiment

A pilot experiment was conducted to determine appropriate amplitudes and frequencies of the pure-tone stimuli used in the main experiments. Ten guinea pigs were used for determining appropriate amplitudes. A click with a duration of 100 µs was used as the stimulus and the recorded signals were averaged across 500 sweeps of alternating polarity stimuli. Sweeps were rejected because of contamination by artifacts when the response amplitude was below -1.5 mA or above 1.5 mA. When the wave III of the EABR was the peak of the resulting waveform, it was assumed that the EABR was evoked successfully; otherwise it was assumed that the amplitude was too low to evoke a measurable EABR. The initial amplitude was set to 70 µA, and it was decreased in steps of 10 µA if a response was recorded, and was increased in steps of 10 µA if no response was recorded. A turn point was defined as occurring when the amplitude changed from increasing to decreasing, or vice versa. After two turn points, the step size was decreased to 5 µA, and after two further turn points it was decreased to 2 µA. The test was stopped after four turn points were obtained at the smallest step size. The threshold estimated as the mean of the amplitude values at the last four turn points. The mean value of the EABR threshold was 54 µA (SD  = 11 µA). The amplitude was fixed at 90 µA for the main experiments except Experiment I.

The other three guinea pigs were used for determining an appropriate frequency of the pure-tone stimuli to be used in measurement of the EFFR. Details of stimulus generation, recording and analysis are given in the sections entitled “Stimulation and recording” and “Calculation of relative amplitude”. The amplitude was fixed at 90 µA, and the frequency of the pure-tone was set to 197, 397, 597, 797, 997, 1597, 2397, or 3997 Hz. These frequencies were selected, because: 1) They cover the frequency range over which the FFR can be recorded [36]; 2) They were shifted by 3 Hz from harmonics of the 50-Hz power-line frequency, hence reducing artifacts. The largest amplitude responses occurred when the frequency was 797 Hz and 1597 Hz. Hence, these two values were chosen for use in the main experiments.

#### 2) I–VII

Before the experiments were started, an EABR test was conducted on every guinea pig to ensure the hearing ability was normal in the same way as described for the pilot experiment. The recorded signals were averaged across 500 sweeps of alternating polarity stimuli. Only guinea pigs whose thresholds were less than 70 µA were used for the main experiments.

In Experiment I, EFFRs were recorded as a function of the stimulus amplitude. The stimulus was an electrical 797-Hz pure tone. The amplitude was varied from 10 µA to 100 µA in steps of 10 µA. The upper limit was fixed at 100 µA, because it was found in the pilot experiment that amplitudes above 110 µA caused muscle twitching. The ten amplitudes were tested in a random order for each guinea pig. The recording time for each amplitude was usually about 5 minutes, and so the experiment took about one hour.

For each guinea pig in group 2, Experiment II–VII were conducted in the following sequence: 1) Gaussian white noise, 797-Hz pure tones, and 1597-Hz pure tones were used as stimuli; 2) Two pure tones (797 Hz and 1597 Hz) with modified envelopes were used as stimuli; 3) Two complex tones with missing F0 were used as stimuli, they were composed of the second, third and fourth harmonics of 797 Hz and 1597 Hz; 4) Pure tones with frequencies of 97, 197, 397, 797, 1597, 3197 and 6397 Hz were presented. The presentation order was random for each guinea pig and different across guinea pigs; 5) A pure tone of 797 Hz was used as the stimulus, and the EFFR was recorded repeatedly every 10 minutes. After it had been recorded three times an overdose of chloral hydrate was injected, and the responses were recorded with the same electrical stimuli 10, 20, 30 minutes later; 6) The guinea pig was replaced by a 1000-ohm electric resistance. Two types of stimuli were used: 797-Hz and 1597-Hz pure tones. The active electrodes from both stimulation and recording instruments were connected to one end of the electric resistance and the reference and ground electrodes were connected to the other end. For all experiments, the recording time for each stimulus condition was usually about 5 minutes, and all experiments could be finished within 2.5 hours.

### Stimulation and recording

**Figure 2:**
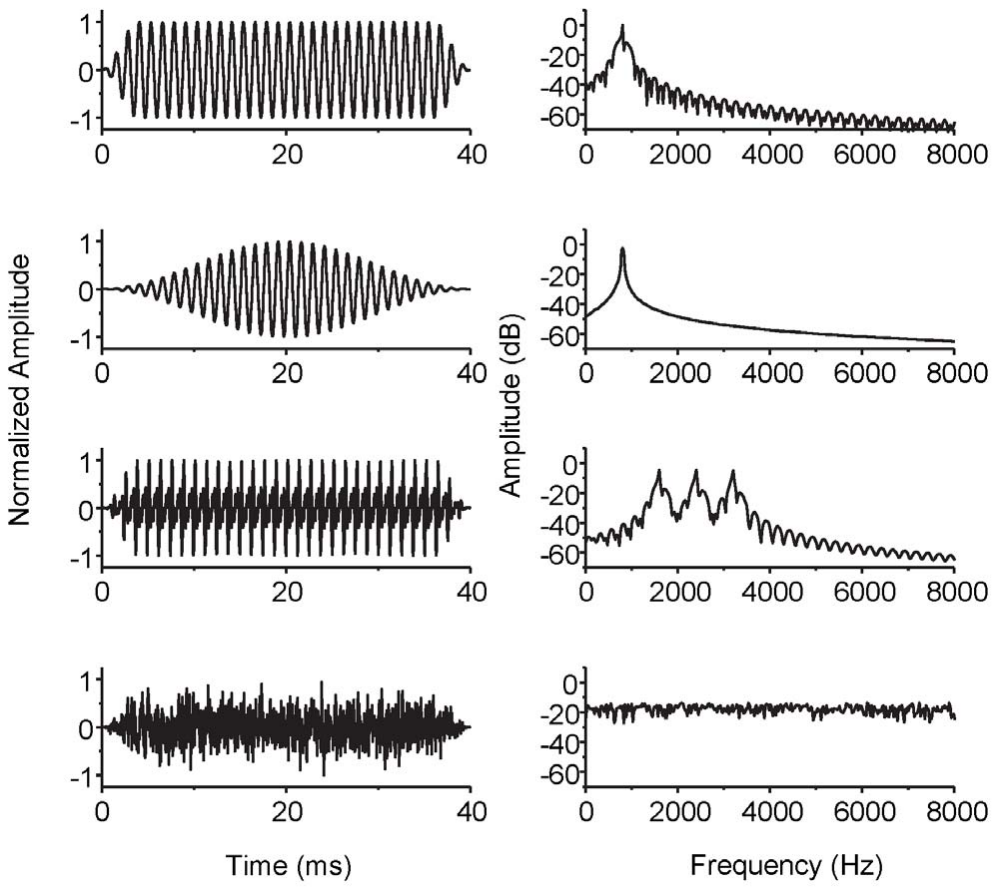
Four types of the stimuli used in this study. From the top to the bottom, the four types of stimuli are represented by the four lines: the 797-Hz pure tone, the 797-Hz pure tone smoothed by a 40-ms Hanning window, the missing fundamental harmonics with F0 = 797 Hz, and the Gaussian white noise. The left column shows the waveforms, and the right column shows the spectra.

All stimuli signals were digitized at 25.6 kHz sampling rate and 16-bit quantization. The waveform and spectrum of the four main types of stimuli are shown in Figure 2. All stimuli were 40-ms long including 4-ms linear onset/offset ramps, except for the signals used in Experiment III, which were gated with a 40-ms Hanning window to make the peak more prominent. Each stimulus was presented with alternating polarity at peak-to-peak value of 180 µA. A total of 1000 valid sweeps containing 500 positive and 500 negative stimuli were presented in pseudo-random order to avoid introducing the effects of stimulus order. A valid sweep was defined as the one whose response amplitude was between -1.5 mA and 1.5 mA, as the response beyond this range was treated as the artifact. This artifact threshold was the same as that used in the EABR tests. During the test, if a sweep was invalid (rejected), the system would automatically resend the stimulating signal till a valid one happened. The percent of valid sweeps was consistently more than 99.5% across all experiments.

The recording system used a 50 kHz sampling rate, 16-bit quantization and a voltage range from -1.5 mV to 1.5 mV. The stimuli were band-pass filtered between 3 and 30000 Hz. The length of the recording window was 100 ms. The repeated rate of the sweeps was 9 Hz to avoid power line inference, and this frequency also avoid aliasing the recording windows (100 ms).

### Data analysis

#### 1) Calculation of relative amplitude

The relative amplitude (*RA*) was used in Experiment II–VII to represent the magnitude of the EFFR. For each stimulus, the recorded signal was averaged across the 1000 sweeps, padded with zeros at the end, and transformed via the Fast Fourier transform (FFT) to twenty five thousands spectral amplitude values. The amplitude over a 100-Hz-wide frequency band centered at the target frequency was used as a measure of *RA* [37], as defined by equation (1):
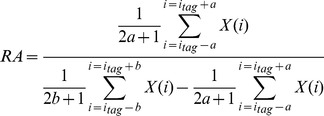
(1)where 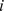 is the index of the bin in the FFT and 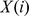 is the FFT amplitude value of the 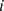 th bin; 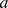 determines the frequency range corresponding to the target frequency, which is set to 1 here, and 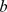 determines the frequency deviation on either side of the target frequency, which is 50 since a 100-Hz-wide frequency band centered at the target frequency is analyzed and frequency resolution is 1 Hz here; 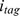 is the index of the target frequency. When the stimulus was a pure tone, the target frequency was the frequency of the pure tone; when the stimulus was a complex tone with missing F0, the target frequency was the value of F0; and when the stimulus was white noise, the target signal was set to that of the comparison pure tone.

#### 2) Estimation of latency

The latency of the EFFR was estimated from the time delay between the peak of the Hanning-windowed signal and the peak in the response.

## Results

### Experiment I: Input-output function

The responses were analyzed in two ways: (1) The average across the 1000 sweeps, including 500 positive and 500 negative stimuli, denoted alternately polarity (AP); (2) The average across 500 positive sweeps, denoted same polarity (SP). The waveform transferred to frequency domain via Fast Fourier transform (FFT) and the amplitude of the target frequency (797 Hz) component was defined as the output amplitude. The input-output function was expressed by calculating the output amplitude in every input amplitude.

**Figure 3:**
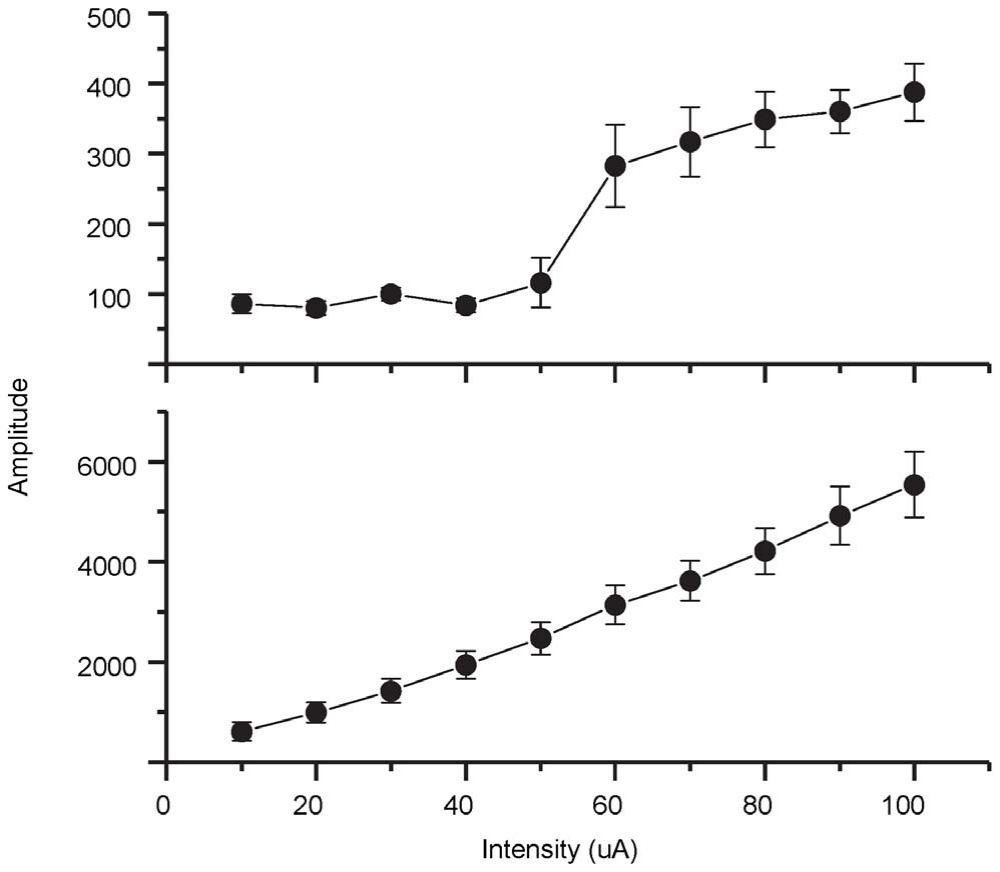
Response amplitudes as a function of the stimulus amplitude in Experiment I. The average output amplitude across 8 guinea pigs is plotted as a function of the input amplitude. The upper panel represents the AP average, and the lower panel represents the SP average. The error bar represents the standard error.

Fig. 3 shows the average output amplitude across 8 guinea pigs as a function of the input amplitude. The output amplitude increased linearly with increasing input amplitude for the SP average, indicating influence of an artifact; however the input-output function was non-linear for the AP average. A linear regression analysis of the amplitude for SP average gave a slope of 55.5 (P<0.001), and an adjusted R^2^ value of 0.994 (P<0.01). For the AP average, a one-way repeated-measures ANOVA with a Greenhouse-Geisser correction showed that main effect of the input amplitude was significant [F(9, 63) = 21.993, P<0.001]. Post hoc tests with Bonferroni correction showed that: (1) The amplitude for each of the three lowest amplitudes (10, 20, 30 µA) was significantly lower than that for each of the three highest amplitudes (80, 90, 100 µA) (P≤0.005<0.05); (2) The difference in amplitude between each pair of the lowest six amplitudes (10, 20, 30, 40, 50, 60 µA) was not significant (P≥0.427>0.05); (3) The difference in amplitude between each pair of the five highest amplitudes (60, 70, 80, 90, 100 µA) is not significant (P≥0.112>0.05); Overall, the results indicate threshold and saturation effects characteristic of neural response rather than artifact.

### Experiment II: Difference of the EFFRs between pure tone and white noise

**Figure 4:**
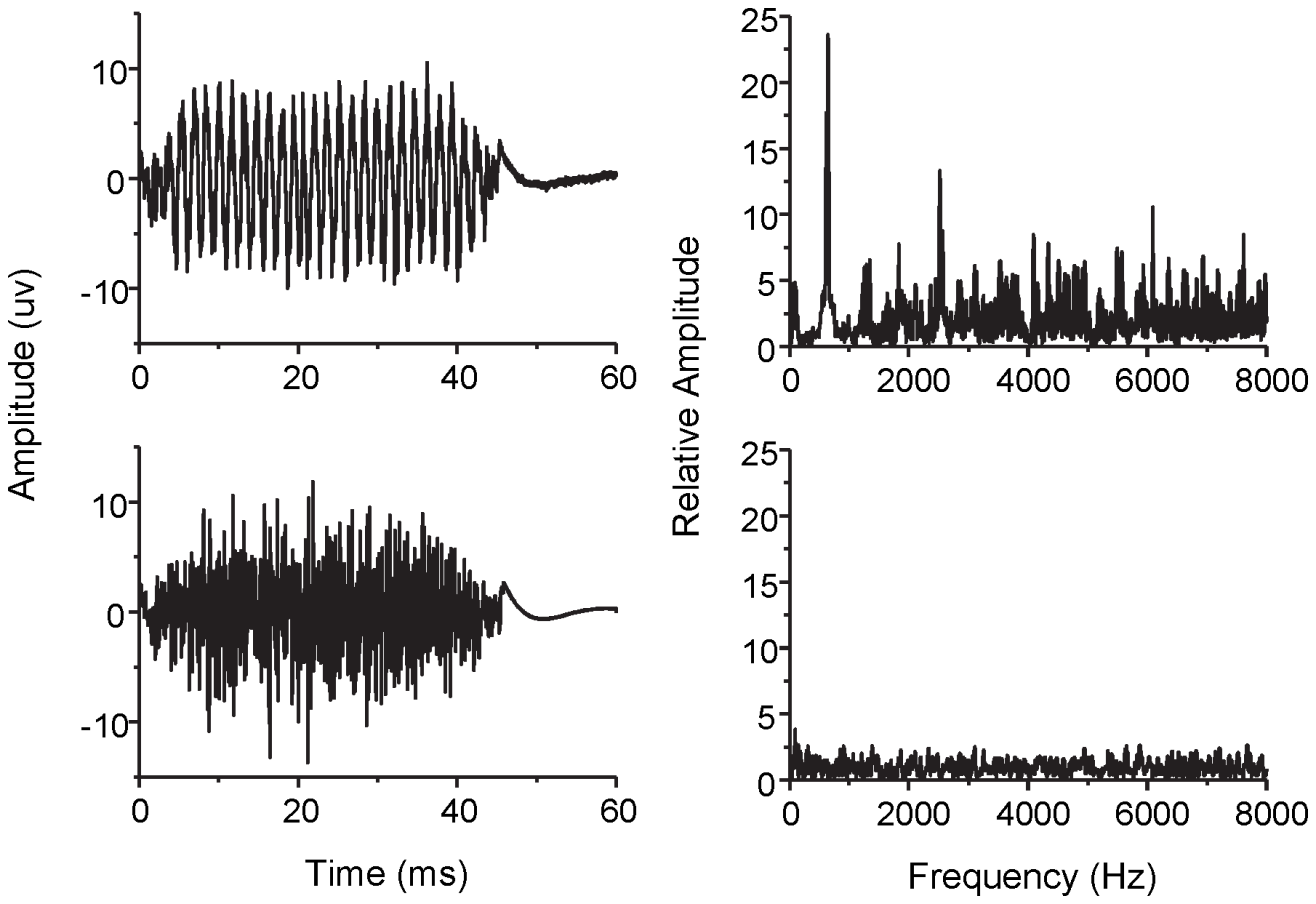
An example of the recorded signals in Experiment II. The recorded signals for the pure tone at 797 Hz (top panels) and Gaussian white noise (bottom panels). The left column shows the waveforms and the right column shows their corresponding relative amplitudes as a function of frequency.

One sample of the recorded signals is shown in Fig. 4. The responses evoked by the pure tone (797 Hz) and by the white noise are shown in the upper and lower panels, respectively. The left column shows the waveforms and the right column shows the *RA* as a function of frequencies. The waveform evoked by the pure tone is periodic, while that evoked by the noise is not. The *RA* for the pure tone shows a prominent peak at 797 Hz, but there is no clear peak for the white noise stimulus.

The average *RA* was 23.0 (SD  = 2.3) for the 797-Hz pure tone and 16.7 (SD  = 0.9) for the 1597-Hz pure tone. For the white noise, the *RA* was 2.2 (SD  = 0.2) at 797 Hz and 2.3 (SD  = 0.2) at 1597 Hz, A two-way (stimulus type and frequency) repeated-measures ANOVA showed a significant effect of the stimulus type [F(1,7) = 266.594, P<0.001], and the effect of frequency just failed to reach significance [F(1,7) = 5.486, P = 0.052>0.05]. These results indicating that the EFFRs can reflect the phase-locked activities in the brainstem, in the same way as FFRs.

### Experiment III: Latencies of the EFFRs

**Figure 5:**
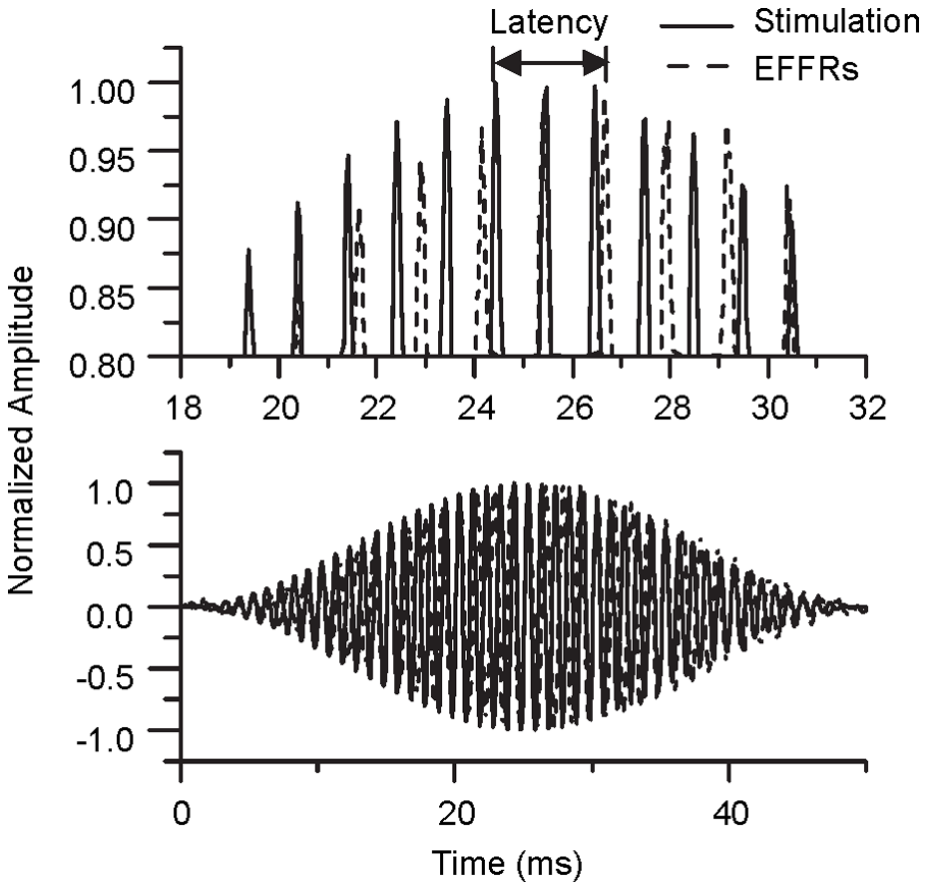
Comparison between the stimulus and response in Experiment III. The bottom panel shows the entire waveforms of the stimulus (solid line) and the response (dotted line) and the top panel shows an expanded view around the peak. The signal was the 797-Hz pure tone.

An example of the temporal alignment of the response with the stimulus is shown in Fig. 5. The lower panel shows the entire waveforms and the upper panel shows a magnified view of the positive peaks between 20 and 30 ms. The latency is about 2.5 ms for this sample. A one-way (stimulus frequency) repeated-measures ANOVA showed that there was no significant difference between the two frequencies [F(1,7) = 0.035, P>0.05].

### Experiment IV: Changes of the EFFR following death

**Figure 6:**
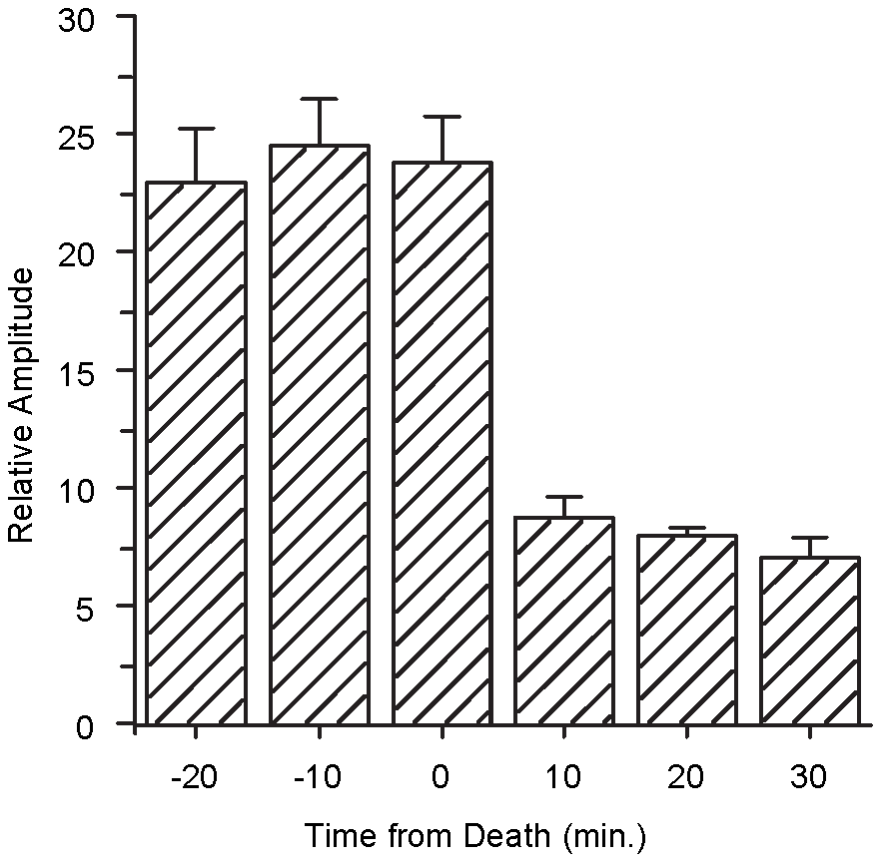
The average relative amplitudes of EFFRs as a function of the time before and after death. All of eight guinea pigs were involved in the statistics and the error bar represents the standard error.

Fig. 6 shows the average *RA* across 8 guinea pigs as a function of time before and after death. There was no clear difference across the three times before death, nor for the three times after death, but the range of the *RA*s was clearly higher for the time before death (22–25) than that for the time after death (6–8). A two-way (before or after death and time interval within each period) repeated-measures ANOVA showed a main effect of life (alive vs. dead) [F(1,7) = 62.842, P<0.001], suggesting that the EFFR is evoked by the neural activity. The main effect of stimulating time was not significant [F(2,21) = 0.736, P>0.05].

### Experiment V: Instrument error analysis by using electrical resistance replacement

**Figure 7:**
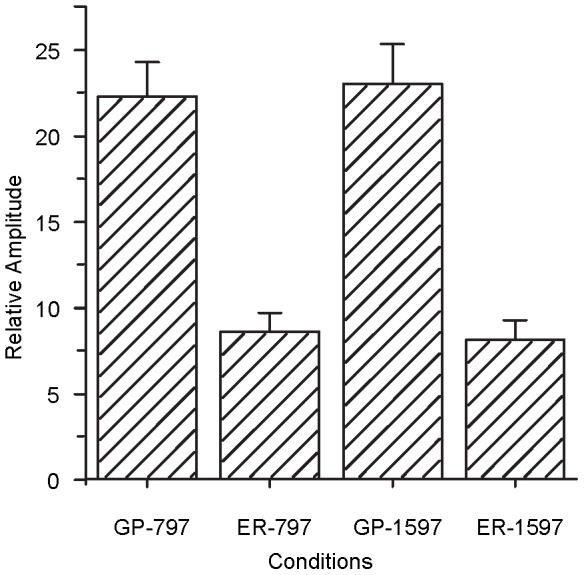
The average relative amplitudes of EFFRs for four different conditions in Experiment V. GP-797 and GP-1597 represent the signal recorded from the guinea pigs (GP) in response to 797-Hz and 1597-Hz pure tones, respectively. ER-797 and ER-1597 represent the signals recorded from the electric resistance (ER) with the same stimuli. All eight guinea pigs were involved in the statistics and the error bar represents the standard error.

The average *RA* recorded on the resistance across 8 repetitions was 8.6 (SD  = 1.1) for the 797-Hz tone and 8.1 (SD  = 1.1) for the 1597-Hz. Figure 7 shows the average *RA* for guinea pigs and electrical resistance in the same stimulus conditions. A two-way (test item and frequency) repeated-measures ANOVA showed a significant effect of test item [F(1,7) = 35.621, P<0.05]. The effect of frequency was not significant [F(1,7) = 0.017, P>0.05].

### Experiment VI: The EFFRs evoked by missing F0 stimuli

**Figure 8:**
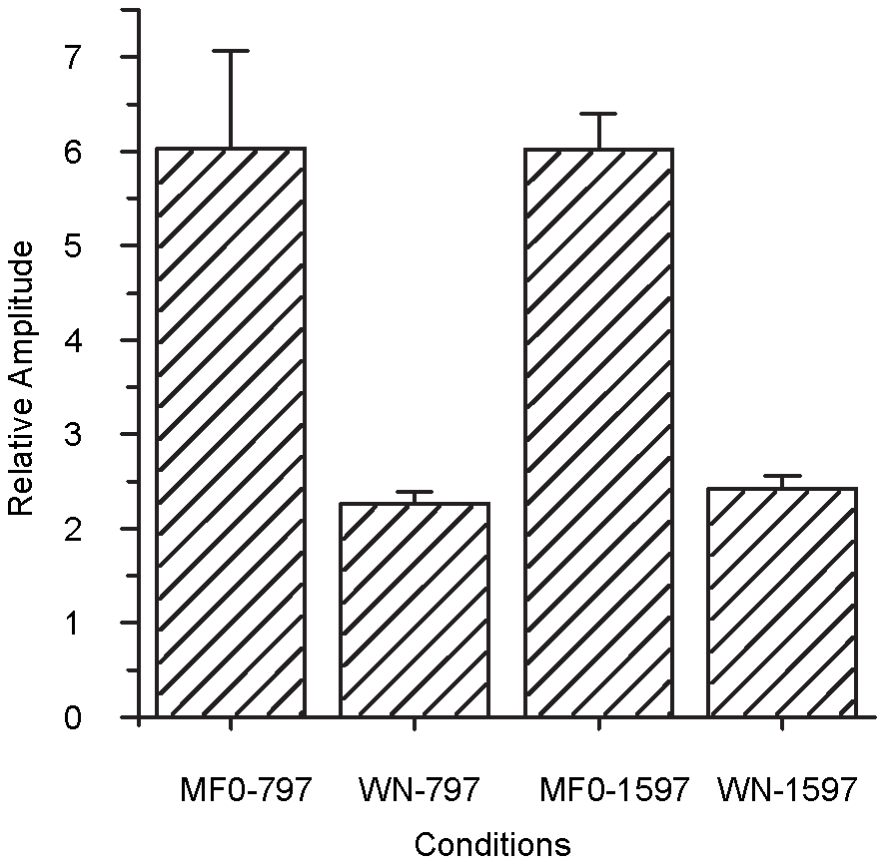
The average relative amplitudes of EFFRs at four different conditions in Experiment VI. MF0-797 and MF0-1597 represent conditions when the stimuli were complex tones with missing F0 797 Hz and 1597 Hz, respectively. WN-797 and WN-1597 represent conditions when the stimulus was white noise. All eight guinea pigs were involved in the statistics and the error bar represents the standard error.

The *RA* at the missing F0 was calculated separately for F0 = 797 Hz and F0 = 1597 Hz. As a control measure, the *RA* was calculated at those two frequencies for the white noise stimulus. Figure 8 shows the average *RA* for the conditions. The average *RA* across 8 guinea pigs was 6.0 (SD  = 1.0) for the 797-Hz tone, and 6.0 (SD  = 0.4) for the 1579-Hz tone. For the white noise, the average *RA* was 2.3 (SD  = 0.1) at 797 Hz, and 2.4 (SD  = 0.1) at 1579 Hz. A two-way (stimulus type and frequency) repeated-measures ANOVA showed a significant effect of the stimulus type [F(1,7) = 44.593, P<0.001], The effect of frequency was not significant [F(1,7) = 0.211, P>0.05]. Although the EFFRs *RA*s evoked by the missing-fundamental stimuli were systematically lower than those evoked by the pure tones, they were still significantly higher than for the control condition, suggesting that EFFRs reflect the phase-locking property of the auditory system for the missing-fundamental stimuli, as has been found in FFR studies using acoustic stimuli [38], [39].

### Experiment VII: The amplitude of EFFRs as a function of the pure-tone frequencies

**Figure 9:**
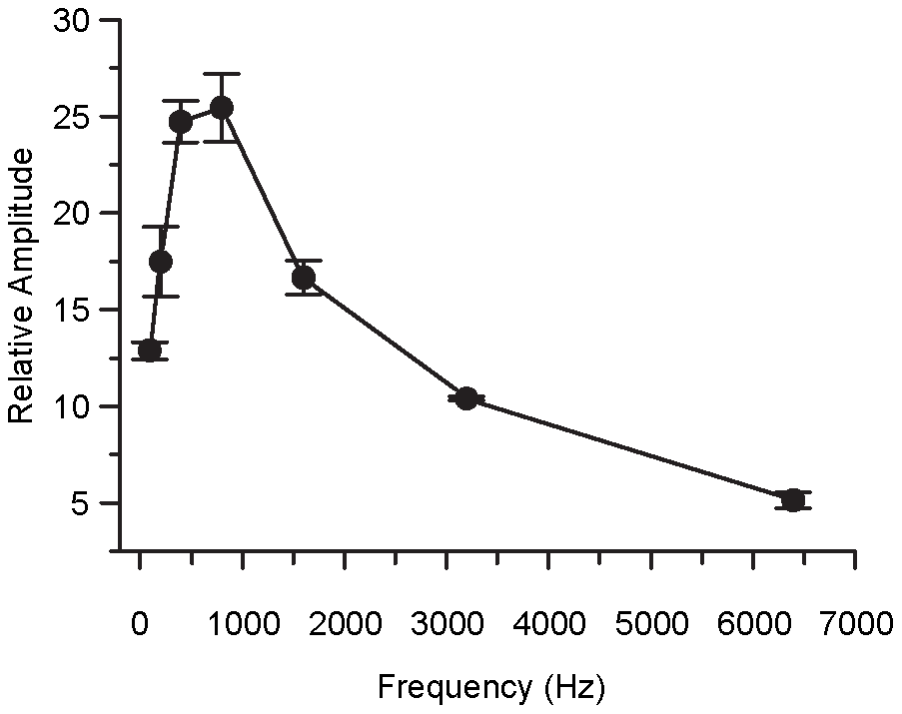
The average relative amplitudes of EFFRs as a function of the pure-tone frequency. All of eight guinea pigs were involved in the statistics and the error bar represents the standard error.

The average *RA*s of the EFFRs as a function of frequency are shown in Fig. 9. The peak value of the *RA* was about 25 for the frequencies of 797 Hz and 397 Hz, indicating that these are suitable frequencies for guinea pigs. This is consistent with FFR studies using acoustic stimuli [36]. The *RA*s of these two frequencies were consistent with the values observed in Experiment II, indicating the repeatability of this EFFR method.

A one-way repeated measure ANOVA with a Greenhouse-Geisser correction showed the main effect of frequency was significant [F(6, 49) = 61.347, P<0.001]. Pairwise t-tests (Bonferroni corrected) indicated that all pair-wide comparisons were significant (t(7)≤−2.710, p<0.01 or t(7)≥3.988, p<0.01) except for the difference between 397 and 797 Hz (t(7) = −0.673, p = 0.523) and the difference between the condition of 197 Hz and 1597 Hz (t(7) = 0.402, p = 0.700).

## Discussion

### EABRs vs. EFFRs

Two broad classes of time-locked responses can be recorded from the brain stem, namely, transient and sustained. As the names suggest, brief, nonperiodic stimulus features evoke transient responses, whereas periodic features elicit sustained phase-locking responses [40]. The EABRs studied in previous publications [12], [14], [41] were all transient responses, evoked by short signals, like clicks with durations between 100–200 µs. However, the EFFR recorded in this study reflected sustained responses evoked by periodic signals, and the experimental results suggest it can reflect the phase-locking response of the auditory system.

Although many of acoustic stimuli have been used in studies of the ABR such as click trains, pure tones, vowels, and even music [40], responses to relatively long-term periodic signals presented in electric form have rarely been presented. Cochlear implants have been considered as the most significant technology in the 21st century for the treatment of severe or profound hearing loss. To determine candidacy, predict the success of cochlear implantation, and provide guidance for improving the implant technology, it is useful be able to know the auditory responses of the electrical stimulation. Using the hardware and software systems provided by cochlear implant manufacturers, it is relatively easy to collect electronically-evoked neural responses [42]. Studies based on EFFR may also supply useful information. However, it should be noted that stimulus-related cochlear implant artifact can sometimes intefere with the measurements in cases of stimulation via a cochlear implant. In many cases, radio frequency pulses (artifact) appear in electrodes near the implant magnet [43], such that the stimulus artifact ends prior to the start of the neural response of interest. Careful consideration of how to avoid this artifact is needed when using the sustained electrical stimulation to measure the EFFR for cochlear implantees.

### Latency calculation

If EFFRs are to be of clinical use for preoperative evaluation, it is important to know where the responses come from. When the origin of the responses is known, the doctor can access which anatomical site is impaired from the abnormal responses. One practicable method for locating the origin is to compare the latency of an unknown response to a known one [44]. Although there is not complete agreement about the origin of each wave peak in EABRs, the common view is that these peaks reflect the neural activities of the eighth nerve and brainstem. In this study the far-field recorded EFFRs have the latency about 2.4 ms which is a bit longer than that of wave III and shorter than that of wave V of the one in the EABRs [36].

However, since the amplitude of EFFR was a function of the stimulus's frequency, and only the frequency of 797 Hz and 1597 Hz were used in Experiment III, the latency evaluation is limited in scope. To address this point, another method [45] was used to evaluate the latency of the EFFR. This method is based on the theory of group delay: any system which responds with a fixed time delay produces a linear phase shift of the response as a function of frequency, and the calculation of the group delay can be calculated by the following equation (2) as follows:
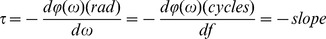
(2)


To calculate the group delay, many samples along the frequency scale are needed. In this experiment, 30 pure-tone frequencies were used. There were 20 frequencies from 297 to 1247 with an interval of 50 Hz and 10 frequencies from 1597 to 2497 with an interval of 100 Hz. The initial phase was always set to zero. All other parameters for producing the stimuli were the same as for the main experiments.

Eight guinea pigs were used for this experiment, and they were treated in the same way for the main experiments. The experimental platform and the recording method were also the same as they were introduced in the previous. The presentation order of the 30 stimuli was random for each guinea pig and different across all guinea pigs.

**Figure 10:**
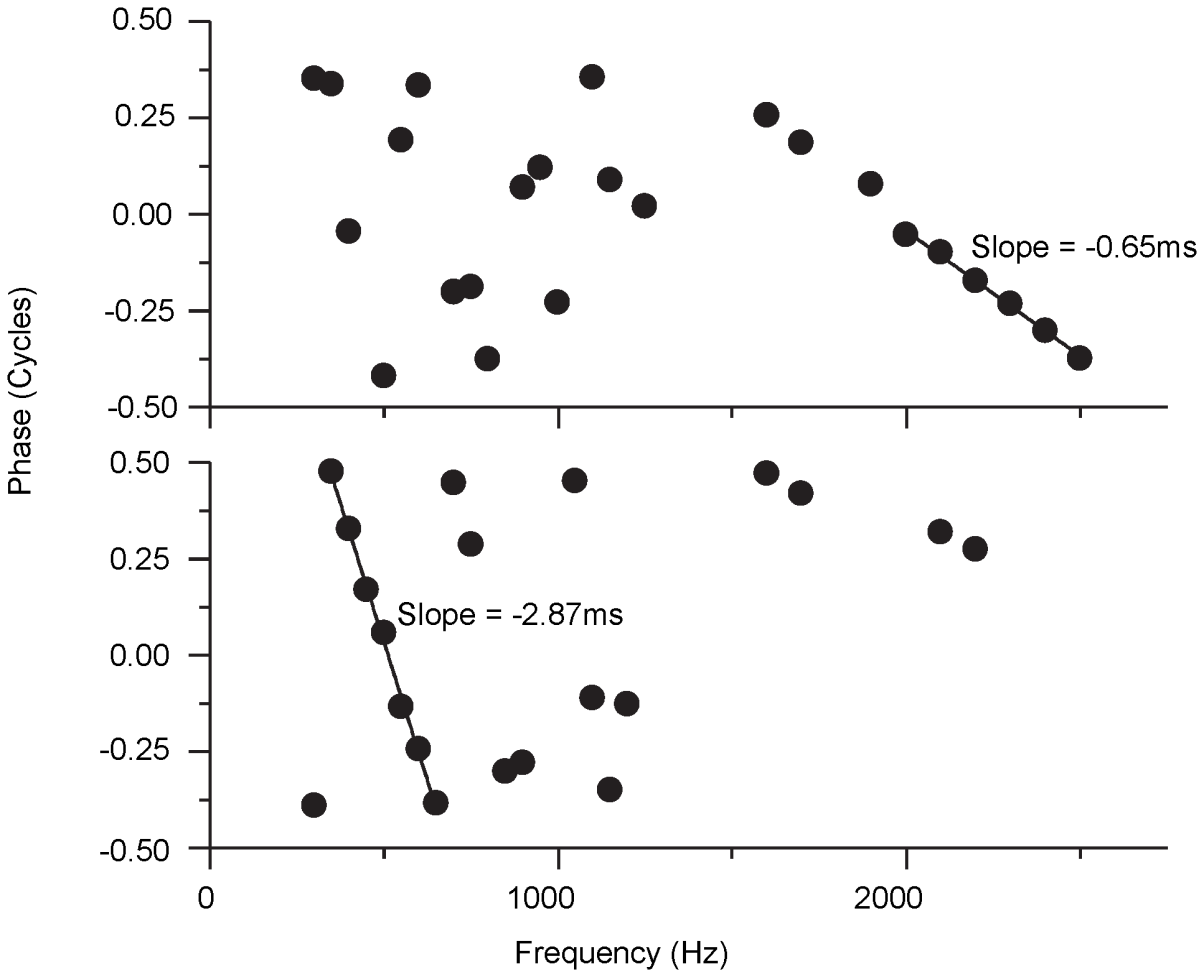
Two examples for calculation of the latencies via group delay. The top panel shows an example where the EFFR group delay was calculated from frequencies between 1900 and 2500 Hz. The bottom panel shows an example where the EFFR group delay was calculated from frequencies between 300 and 700 Hz.

The amplitude and phase were calculated by FFT separately for each recorded signal. Some invalid data were deleted before calculating the latency. The criterion for deletion was estimated by calculating spectral amplitudes at the frequencies near that of the stimulus (target frequency). These amplitudes represent the noise in the area of the target frequency, and their mean and standard deviation were calculated. Only the recorded signals for which the amplitude of the target frequency component was greater than the mean plus 5 standard deviations were treated as the valid data [45]. After deleting the noisy data, a least-squares fitting method was used to fit the scattered phase data over a suitable range (see Fig. 10), that was defined as a range where the phase varied monotonically across 6 or more adjacent frequencies.

The group delay can be calculated for 6 of the 8 guinea pigs. Two samples of the data for the 6 guinea pigs are shown in Fig. 10. For the sample in the upper panel, the group delay was 0.65 ms, which was considered as an outlier, as it was unreasonable in principle. A similar phenomenon was described in [45], and it was considered that it was caused by cochlear microphonic. Similar latencies (group delays) were founded across the remaining 5 guinea pigs. The mean value was 2.8 ms (SD  = 0.2 ms). The data used for fitting the group delay were all located in the area of frequency range 297–1947 Hz for these 5 guinea pigs.

The latency measured by group delay here was similar to the latency tested in Experiment III (about 2.4 ms). The latencies of the ABR for the young guinea pigs reported in [46] were approximately 0.8–1.6 ms, 16–2.4 ms, 2.4–3.1 ms and 3.2–3.8 ms for waves I– IV. The latencies decreased with increasing amplitude. Electrically stimuli often usually lead to a shorter latency than acoustic stimuli [41]. The latency of FFR reported in previous study was increased from 2.5 ms to 5.3 ms as the amplitude of stimuli decreased from a high level to the threshold [47]. From the input-output function (Fig. 3), the mean threshold of the EFFR for these 8 guinea pigs was around 60 µA. So the latency reported in this article was reasonably consistent with that reported in previous studies considering the effect of amplitude and the differences between electrically stimuli and acoustic stimuli. And the EFFRs recorded above may be originated from cochlear nucleus and/or lateral lemniscus due to the range of the latencies and pervious study in cats [19].

### Other topics

FFRs evoked by harmonic stimuli with missing F0 have been studied previously [39]. Comparing of the results of Experiment V and Experiment I, it showed that the response at the missing F0 was smaller than the response to pure tones at the same frequencies. This difference is consistent with that found for FFRs [37], and it is probably caused by the different machenisms for coding the two types of stimuli in the auditory system. The frequency of the pure tone signal is directly represented in the patterns of phase locking in the auditory nerve, but the missing F0 is not directly coded in the peripheral neural response,when only low (resolved) harmonics are present.

When the frequency of pure tone was manipulated in Experiment VII, the results indicated that the responses were bigger for 397 and 797 Hz than for other frequencies, and the response was weak when the frequency was 3197 Hz or above. This pattern is consistent with a previous study, which reported that the upper limit of phase-locking in the cochlear nerve was about 3500 Hz, and the strength of phase-locking fell off between 500 and 1000 Hz [48].

Although the EFFR can be recorded on guinea pigs, there are several problems to be concerned for implementing the similar experiments on human. The EFFR test spent more time than the EABR due to their difference on the duration of stimulating signal and the sweep number (40 ms vs. 100 µs for each sweep; 1000 sweeps vs. 500 sweeps in this study), which might increase the risk of anesthesia and surgery operation for placing the electrodes in human. Furthermore, for the EABR measure on human, the position of the electrode placement, the parameters of electric pulses, and the relationship between EABRs and psychophysical perception have been studied a lot, but these issues remain unknown for the EFFR measure. These topics clearly need further work and discussion.

In summary the EFFRs method has been shown to reflect neural responses rather than artifacts. Further research is needed to determine the difference between thresholds measured using EFFRs and EABRs both preoperative and postoperative, to determine the influence on electrode placement of EFFRs, and to determine the effect of modifying parameter setting of the coding strategies used in cochlear implants based on the postoperative EFFRs.

## References

[1] Katz J, Medwetsky L, Burkard R, Hood L (2010) Handbook of clinical audiology: Lipinicott Williams & Wilkins.

[2] Moore DR, Shannon RV (2009) Beyond cochlear implants: Awakening the deafened brain. Nat Neurosci 12: 686–691. 19471266 10.1038/nn.2326

[3] Burkard RF, Eggermont JJ, Don M (2007) Auditory evoked potentials: basic principles and clinical application: Lippincott Williams & Wilkins.

[4] Polak M, Eshraghi AA, Nehme O, Ahsan S, Guzman J, et al (2004) Evaluation of hearing and auditory nerve function by combining ABR, DPOAE and eABR tests into a single recording session. J Neurosci Methods 134: 141–149. 15003380 10.1016/j.jneumeth.2003.11.003

[5] Jewett LD, Williston SJ (1971) Auditory-evoked far fields averaged from the scalp of humans. Brain 94: 681–696. 5132966 10.1093/brain/94.4.681

[6] Achor LJ, Starr A (1980) Auditory brain stem responses in the cat. I. Intracranial and extracranial recordings. Electroencephalogr Clin Neurophysiol 48: 154–173. 6153332 10.1016/0013-4694(80)90301-6

[7] Wada S-I, Starr A (1983) Generation of auditory brain stem responses (ABRs). III. Effects of lesions of the superior olive, lateral lemniscus and inferior colliculus on the ABR in guinea pig. Electroencephalogr Clin Neurophysiol 56: 352–366. 6193949 10.1016/0013-4694(83)90261-4

[8] Møller AR, Burgess J (1986) Neural generators of the brain-stem auditory evoked potentials (BAEPs) in the rhesus monkey. Electroencephalogr Clin Neurophysiol 65: 361–372. 2427327 10.1016/0168-5597(86)90015-8

[9] Starr A (1976) Correlation between confirmed sites of neurological lesions and abnormalities of far-field auditory brainstem responses. Electroencephalogr Clin Neurophysiol 41: 595–608. 62654 10.1016/0013-4694(76)90005-5

[10] Mason JAH (1998) Universal infant hearing screening by automated auditory brainstem response measurement. Pediatrics 101: 221–228. 9445495 10.1542/peds.101.2.221

[11] Gibson WPR, Graham JM (2008) Editorial: ‘Auditory neuropathy’ and cochlear implanatation - myths and facts. Cochlear Implants Int 9: 1–7. 18246533 10.1179/cim.2008.9.1.1

[12] Starr A, Brackmann DE (1978) Brain stem potentials evoked by electrical stimulation of the cochlea in human subjects. Ann Otol Rhinol Laryngol 88: 550–556. 10.1177/000348947908800419 475255

[13] Simmons FB, Lusted HS, Meyers T, Shelton C (1984) Electrically induced auditory brainstem response as a clinical tool in estimating nerve survival. Ann Otol Rhinol Laryngol Supp 112: 97–100. 10.1177/00034894840930s417 6431890

[14] Kileny PR, Zwolan TA (2004) Pre-perioperative, transtympanic electrically evoked auditory brainstem response in children. Int J Audiol 43 Suppl 1 S16–21. 15732377

[15] Wang L, Zhang Q, Wang Q, Dong M, Zeng Y (2009) Functional evaluation of auditory system in patients with cochlear implant using electrically evoked auditory brainstem responses. Acoust Phys 55: 857–865.

[16] Moore BCJ (2012) An introduction to the psychology of hearing: BRILL.

[17] Evans EF (1975) Cochlear Nerve and Cochlear Nucleus. In: Keidel WD, Neff WD, editors. Auditory System: Springer Berlin Heidelberg. pp.1–108.

[18] Stillman RD, Crow G, Moushegian G (1978) Components of the frequency-following potential in man. Electroencephalogr Clin Neurophysiol 44: 438–446. 76552 10.1016/0013-4694(78)90028-7

[19] Gardi J, Merzenich M, McKean C (1979) Origins of the scalp-recorded frequency-following response in the cat. Int J Audiol 18: 353–380. 496719

[20] Chandrasekaran B, Kraus N (2010) The scalp-recorded brainstem response to speech: Neural origins and plasticity. Psychophysiology 47: 236–246. 19824950 10.1111/j.1469-8986.2009.00928.x PMC3088516

[21] Du Y, Kong LZ, Wang Q, Wu XH, Li L (2011) Auditory frequency-following response: A neurophysiological measure for studying the cocktail-party problem. Neurosci Biobehav Rev 35: 2046–2057. 21645541 10.1016/j.neubiorev.2011.05.008

[22] Johnson KL, Nicol TG, Kraus N (2005) Brain stem response to speech: A biological marker of auditory processing. Ear Hear 26: 424–434. 16230893 10.1097/01.aud.0000179687.71662.6e

[23] Aiken SJ, Picton TW (2008) Envelope and spectral frequency-following responses to vowel sounds. Hear Res 245: 35–47. 18765275 10.1016/j.heares.2008.08.004

[24] Akhoun I, Gallégo S, Moulin A, Ménard M, Veuillet E, et al (2008) The temporal relationship between speech auditory brainstem responses and the acoustic pattern of the phoneme/ba/in normal-hearing adults. Clin Neurophysiol 119: 922–933. 18291717 10.1016/j.clinph.2007.12.010

[25] Wever EG, Bray CW (1930) Action currents in the auditory nerve in response to acoustic stimulation. Proc Natl Acad Sci U S A 16: 344–350. 16587578 10.1073/pnas.16.5.344 PMC526644

[26] Derbyshire AJ, Davis H (1935) The action potentials of the auditory nerve. Am J Physiol 113: 476–504.

[27] Moushegian G, Rupert A, A M (1964) Brain-stem neuronal response patterns to monaural and binaural tones. J Neurophysiol 27: 1174–1191. 14223977 10.1152/jn.1964.27.6.1174

[28] Marsh JT, Worden FG, Smith JC (1970) Auditory frequency-following response: neural or artifact? Science 169: 1222–1223. 5450700 10.1126/science.169.3951.1222

[29] Moushegian G, Rupert AL, Stillman RD (1973) Scalp-recorded early responses in man to frequencies in the speech range. Electroencephalogr Clin Neurophysiol 35: 665–667. 4128165 10.1016/0013-4694(73)90223-x

[30] Gerken GM, Moushegian G, Stillman RD, Rupert AL (1975) Human frequency-following responses to monaural and binaural stimuli. Electroencephalogr Clin Neurophysiol 38: 379–386. 46818 10.1016/0013-4694(75)90262-x

[31] Smith JC, Marsh JT, Brown WS (1975) Far-field recorded frequency-following responses: Evidence for the locus of brainstem sources. Electroencephalogr Clin Neurophysiol 39: 465–472. 52439 10.1016/0013-4694(75)90047-4

[32] Marsh JT, Worden FG (1968) Sound evoked frequency-following responses in the central auditory pathway. Laryngoscope 78: 1149–1163. 5659588 10.1288/00005537-196807000-00003

[33] Xiong M, He Q, Lai H, Wang J (2011) Oxidative stress in spiral ganglion cells of pigmented and albino guinea pigs exposed to impulse noise. Acta Otolaryngo 131: 914–920. 10.3109/00016489.2011.577448 21542672

[34] Harrison RV, Palmer A, Aran J-M (1984) Some otological differences between pigmented and albino-type guinea pigs. Arch Oto-Rhino-Laryn 240: 271–275. 10.1007/BF00453382 6487138

[35] Tapp R, Eliel M, Dolan DD, Altschuler RA, Gauvin DV, et al (2009) Comparison of pigmented and albino guinea pigs for use in ototoxicity modeling. J Pharmaclo Toxicol 60: 231.

[36] Ping JL, Li NX, Galbraith GC, Wu XH, Li L (2008) Auditory frequency-following responses in rat ipsilateral inferior colliculus. Neuroreport 19: 1377–1380. 18766015 10.1097/WNR.0b013e32830c1cfa

[37] Du Y, Ma T, Wang Q, Wu X, Li L (2009) Two crossed axonal projections contribute to binaural unmasking of frequency-following responses in rat inferior colliculus. Eur J Neurosci 30: 1779–1789. 19840111 10.1111/j.1460-9568.2009.06947.x

[38] Smith JC, Marsh JT, Greenberg S, Brown WS (1978) Human auditory frequency-following responses to a missing fundamental. Science 201: 639–641. 675250 10.1126/science.675250

[39] Galbraith GC (1994) Two-channel brain-stem frequency-following responses to pure tone and missing fundamental stimuli. Electroencephalogr Clin Neurophysiol 92: 321–330. 7517854 10.1016/0168-5597(94)90100-7

[40] Skoe E, Kraus N (2010) Auditory brianstem response to complex sounds: a tutorial. Ear Hear 31: 302–324. 20084007 10.1097/AUD.0b013e3181cdb272 PMC2868335

[41] van den Honert C, Stypulkowski PH (1986) Characterization of the electrically evoked auditory brainstem response (ABR) in cats and humans. Hear Res 21: 109–126. 3754550 10.1016/0378-5955(86)90033-x

[42] Miller CA, Brown CJ, ABbas PJ, Chi SL (2008) The clinical application of potentials evoked from the peripheral auditory system. Hear Res 242: 184–197. 18515023 10.1016/j.heares.2008.04.005

[43] Martin BA (2007) Can the acoustic change complex be recorded in an individual with a cochlear implant? Separating neural responses from cochlear implant artifact. J Am Acad Audiol 18: 126–140. 17402299 10.3766/jaaa.18.2.5

[44] Stillman RD, Moushegian G, Rupert A (1976) Early Tone-Evoked Responses in Normal and Hearing-Impaired Subjects. Int J Audiol 15: 10–22. 10.3109/00206097609071760 1252187

[45] Batra R, Kuwada S, MaherV L (1986) The frequency-following response to continuous tones in humans. Hear Res 21: 167–177. 3700255 10.1016/0378-5955(86)90037-7

[46] Ingham NJ, Thornton SK, Comis SD, Withington DJ (1998) The auditory brainstem response of aged guinea pigs. Acta Otolaryngo 118: 673–680. 10.1080/00016489850183160 9840503

[47] Dum N, Schmidt U, Vonwedel H (1982) Frequency-dependence of early auditory evoked responses in the guinea pig. Arch Oto-Rhino-Laryn 236: 59–66. 10.1007/BF00464058 7126031

[48] Liu LF, Palmer AR, Wallace MN (2006) Phase-locked responses to pure tones in the inferior colliculus. J Neurophysiol 95: 1926–1935. 16339005 10.1152/jn.00497.2005

